# Vertebral marrow fat fraction is associated with circulating RANKL in postmenopausal females

**DOI:** 10.3389/fendo.2024.1442046

**Published:** 2024-09-16

**Authors:** Xuefeng Li, Xiaoyong Zuo, Li Lu, Run Xu, Ying Wang, Shixin Chang, Yi Wang, Peng Luo, Guanwu Li

**Affiliations:** ^1^ Department of Radiology, Yueyang Hospital of Integrated Traditional Chinese and Western Medicine, Shanghai University of Traditional Chinese Medicine, Shanghai, China; ^2^ Department of Clinical Laboratory, Shanghai Fourth People’s Hospital, School of Medicine, Tongji University, Shanghai, China; ^3^ Department of Gastroenterology, Yueyang Hospital of Integrated Traditional Chinese and Western Medicine, Shanghai University of Traditional Chinese Medicine, Shanghai, China

**Keywords:** menopause, marrow adipose tissue, RANKL, bone mineral density, osteoprotegerin

## Abstract

**Objective:**

To investigate the relationship between circulating receptor activator of nuclear factor-kappa B ligand (RANKL) levels and marrow adipose tissue in postmenopausal females.

**Methods:**

A total of 164 postmenopausal females were included in the study. Serum levels of osteoprotegerin (OPG) and RANKL were measured using ELISA kits. Body composition and bone mineral density (BMD) were assessed using dual-energy X-ray absorptiometry. Complex-based chemical shift imaging-based MRI was employed to evaluate the vertebral marrow proton density fat fraction (PDFF). A multivariate linear regression model was utilized to analyze the predictive effects of PDFF and BMD on circulating levels of OPG and RANKL.

**Results:**

Simple regression analysis showed significant associations among the marrow PDFF, BMD at either site, serum RANKL, and the RANKL/OPG ratio. In multivariate linear regression models, marrow PDFF was found to have a positive correlation (β = 3.15, 95% CI 2.60 to 3.70) and BMD had negative correlations (β = −0.200, 95% CI −0.348 to −0.051 for vertebral BMD; β = −0.383, 95% CI −0.589 to −0.177 for total hip BMD; and β =−0.393, 95% CI −0.598 to −0.188 for femoral neck BMD, all p < 0.01) with circulating soluble RANKL levels after adjusting for age, body mass index, physical activity, total fat mass, android/gynoid ratio, and lean mass. Similar results were observed for the RANKL/OPG ratio. Additionally, multivariate linear regression analyses revealed that marrow PDFF was a significant independent contributor of circulating soluble RANKL (β = 1.34, 95% CI 1.10 to 1.58, p < 0.001) after further controlling for BMD. However, marrow PDFF or BMD had no associations with circulating levels of OPG after adjusting for all potential confounders mentioned above.

**Conclusions:**

Vertebral marrow fat fraction is independently associated with circulating soluble RANKL levels in postmenopausal females.

## 1 Introduction

Receptor activator of nuclear factor-kappa B ligand (RANKL) and its cognate receptor, activator of nuclear factor kappa B (RANK), are crucial for the differentiation, survival, and activity of osteoclasts (1). RANKL, expressed by osteoblasts and osteocytes, binds to RANK on the membrane of osteoclast progenitors. Besides osteoblasts and osteocytes, other cells such as immune cells and hypertrophic chondrocytes also produce RANKL (2). Osteoprotegerin (OPG) is a soluble decoy receptor for RANKL that inhibits the RANKL-RANK interaction, thereby mediating osteoclastogenesis. The RANK/RANKL/OPG signaling pathways are vital for bone resorption (3). Therefore, measuring the levels of RANKL, OPG, and their ratio can provide insights into bone formation and resorption.

Previous studies have shown that circulating levels of soluble RANKL are elevated in animal models of menopause (4, 5) and in mice with postmenopausal obesity induced by ovariectomy and subsequent high-fat and high-sucrose diet feeding (1). Several observational studies have attempted to determine the relationships between circulating RANKL levels, OPG levels, the RANKL/OPG ratio, and bone mineral density (BMD) (6–11), but the results have been inconsistent. Bone marrow adipose tissue, which constitutes more than 10% of total body fat, has been implicated in various physiological and pathological conditions, including osteoporosis, diabetes, and certain cancers (12–14). Aging is associated with a decline in BMD and a significant increase in marrow adipose tissue. Increased marrow adiposity is linked to decreased osteoblast numbers and bone formation, increased bone resorption, and osteoporotic pathologies characterized by altered bone structure, reduced bone mass, and increased fracture risk (15–17). In animal models, enhanced bone resorption in early-stage type I diabetes has been attributed to RANKL derived from marrow fat rather than from bone tissue itself (18). Recent ex vivo and animal studies have also suggested that adipose lineage cells within the bone marrow express RANKL (19–22). Therefore, bone marrow adipose tissue may be an important factor influencing soluble RANKL levels.

However, little is known about the relationships between bone marrow fat content, serum soluble RANKL levels, and the RANKL/OPG ratio in humans. This is a significant knowledge gap because the interactions between bone marrow fat and the RANKL/OPG system in bone balance are not well understood. Hence, the aim of our study was to investigate the associations between circulating soluble RANKL levels, OPG levels, and the RANKL/OPG ratio with body composition and marrow adipose tissue, as assessed by dual-energy X-ray absorptiometry and complex-based chemical shift imaging-based MRI, in postmenopausal females.

## 2 Methods

### 2.1 Study participants

The study, conducted between March 2019 and June 2023, recruited 176 postmenopausal females aged 50 to 87 from the community. Participants met the following inclusion criteria: they were ambulatory, aged over 50, had experienced menopause at least one year prior (defined as amenorrhea for at least 12 months after the last menstrual period) (12), and were capable of providing informed consent. The exclusion criteria were comprehensive (1): Presence of other metabolic or inherited bone diseases, such as Paget’s disease, hyperparathyroidism, hypoparathyroidism, or vitamin D deficiency (2); Endocrine disorders impacting bone or fat metabolism, including hyperthyroidism and diabetes mellitus (3); Cardiovascular diseases, chronic liver or kidney diseases, chronic lung diseases, history of malignancy, autoimmune diseases, rheumatic inflammatory diseases, and depressive disorders (4); Current or past use of medications affecting bone marrow metabolism, such as glucocorticoids, bisphosphonates, aromatase inhibitors, anticonvulsant therapy, hormone replacement therapy, and lipid-lowering agents (5); Neurological diseases or cerebrovascular accident sequelae affecting the musculoskeletal system (6); Vitamin D supplementation exceeding 1200 IU daily, alcohol consumption, current smoking, or a body mass index (BMI) outside the range of 19-35 kg/m². Twelve participants were excluded due to metastasis (n = 1), hyperglycemia (n = 5), and silent vertebral body fracture (n = 6), resulting in a final sample of 164 participants.

A standardized physical examination was conducted for all participants, with data recorded on age, menopausal status, body weight, height, drinking habits, alcohol consumption, smoking history, and physical activity. BMI was calculated from body weight and height. Physical activity was defined as moderate activity occurring at least three times per week for at least 30 minutes (23). The research protocol was approved by the institute’s Ethics Committee (no. 2018-075), adhering to the ethical principles of the Declaration of Helsinki and its amendments. Written informed consent was obtained from all participants.

### 2.2 Laboratory analyses

Morning blood samples for laboratory analysis were collected from all participants between 7:00 and 10:00 AM after an 8-hour overnight fast. Lipid profiles (total cholesterol, triglycerides, high-density lipoprotein cholesterol, and low-density lipoprotein cholesterol) and plasma fasting glucose were analyzed using a chemiluminescence immunoassay system. Enzymatic methods were employed to measure serum calcium, phosphorus, creatinine and uric acid levels.

The estimated glomerular filtration rate was calculated utilizing the Chronic Kidney Disease Epidemiology Collaboration equation. Serum levels of bone turnover biomarkers, including 25-hydroxyvitamin D, parathyroid hormone, β-type I collagen telopeptides, osteocalcin, and N-terminal propeptide of type 1 procollagen, were determined using an electrochemiluminescence immunoassay system (Cobas 8000 e801; Roche Diagnostics, Basel, Switzerland). The intra-assay and inter-assay coefficients of variation (CVs) for these biomarkers ranged from 3.2% to 7.0%. Serum levels of RANKL and OPG were measured by enzyme-linked immunosorbent assay (MULTISCIENCES, Hangzhou, China), with values obtained from standard curves in duplicate according to the manufacturer’s instructions. The intra-assay and inter-assay CVs for soluble RANKL and OPG were less than 8.6% and 7.7%, respectively.

### 2.3 BMD and body composition measurements

Dual-energy X-ray absorptiometry (Prodigy Lunar; GE Healthcare, Waukesha, WI) was used to determine total and regional bone and soft tissue compositions, including total fat mass, total lean mass, and android and gynoid fat amounts. BMD (g/cm²) was measured at the total hip, femoral neck, and lumbar spine (L1-L4). The CVs for BMD measurements were less than 1.3% for the lumbar spine, 1.8% for the femoral neck, and 1.4% for the total hip. Additionally, the CVs for total lean mass and fat mass were 0.73% and 1.0%, respectively.

### 2.4 MR image acquisition and data analysis

All imaging was performed on a clinical 3.0-T whole-body MR imager (uMR 780, United Imaging Healthcare). All participants underwent MRI examination of the lumbar spine on the same day as the dual-energy X-ray absorptiometry scan. The interval between laboratory analysis and image acquisitions was within one week. To identify pre-existing abnormalities of the lumbar vertebrae, routine MR imaging of the lumbar spine was performed using sagittal T1-weighted and T2-weighted sequences, as well as a transverse T2-weighted sequence.

To determine the percentage PDFF, which is the ratio of fat to the sum of water and fat, a commercially available 3D spoiled gradient echo pulse sequence called Fat Analysis & Calculation Technique (FACT) was used. The sequence had equidistant echo spacing of 1.1 ms, with the first TE at 1.21 ms. The TR was set to the shortest possible duration for the 6-echo FACT, which is 7.2 ms. A low flip angle of 3° was applied to minimize T1 saturation effects, and a multipeak fat model was used to address the spectral complexity of fat (24). Parallel imaging was employed with a SENSE factor of 2 in the anterior-posterior direction. The total scan time was 17.5 seconds, completed in a single breath-hold. Additional imaging parameters included a receiver bandwidth of 1.3 kHz, a field of view of 40 cm, an acquisition matrix size of 256 x 192, a slice thickness of 3 mm, no interslice gap, and 1 average.

FACT images were transferred to the uWS-MR Advanced Postprocess Workstation (United Imaging Healthcare), where the parametric PDFF maps were automatically generated. The CV of PDFF measured by FACT in the lumbar spine of ten subjects, who were scanned twice on the same day after repositioning, was 2.8%.

### 2.5 Statistical analysis

Statistical analysis was performed using SPSS (version 27.0; Chicago, IL, USA) and GraphPad Prism 10. Results were expressed as mean ± standard deviation (SD) for continuous variables, median (interquartile range [IQR]) for non-normally distributed continuous variables, and n (%) for categorical variables. The normality of distributions was assessed using the Shapiro-Wilk test. Trends in marrow PDFF across soluble RANKL, OPG, and their ratio quartiles were tested using a polynomial test. Least square mean values of marrow PDFF were compared across quartiles of circulating RANKL, OPG, or the RANKL/OPG ratio, controlling for age, BMI, physical activity (moderate intensity), total fat mass, android/gynoid ratio, and lean mass. Multiple linear regression analysis was conducted to test for linear trends. Pearson’s and Spearman’s correlations were used to investigate bivariate associations between variables for normally and non-normally distributed data, respectively. A multivariate linear regression model was used to investigate the independent effect of BMD (at the lumbar spine, total hip, and femoral neck) on circulating RANKL, OPG, or the RANKL/OPG ratio, adjusting for the potential covariates mentioned above. A similar analysis was conducted to explore the correlation between marrow PDFF and serum RANKL, OPG, and the RANKL/OPG ratio. To avoid multicollinearity in the regression model, tolerance and variance inflation factor (VIF) were evaluated. A tolerance value less than 0.3 and a variance inflation factor value above 2 indicated potential issues with the model (25). The level of significance was set at 0.05.

## 3 Results

### 3.1 Characteristics of the study participants

**Figure 1:**
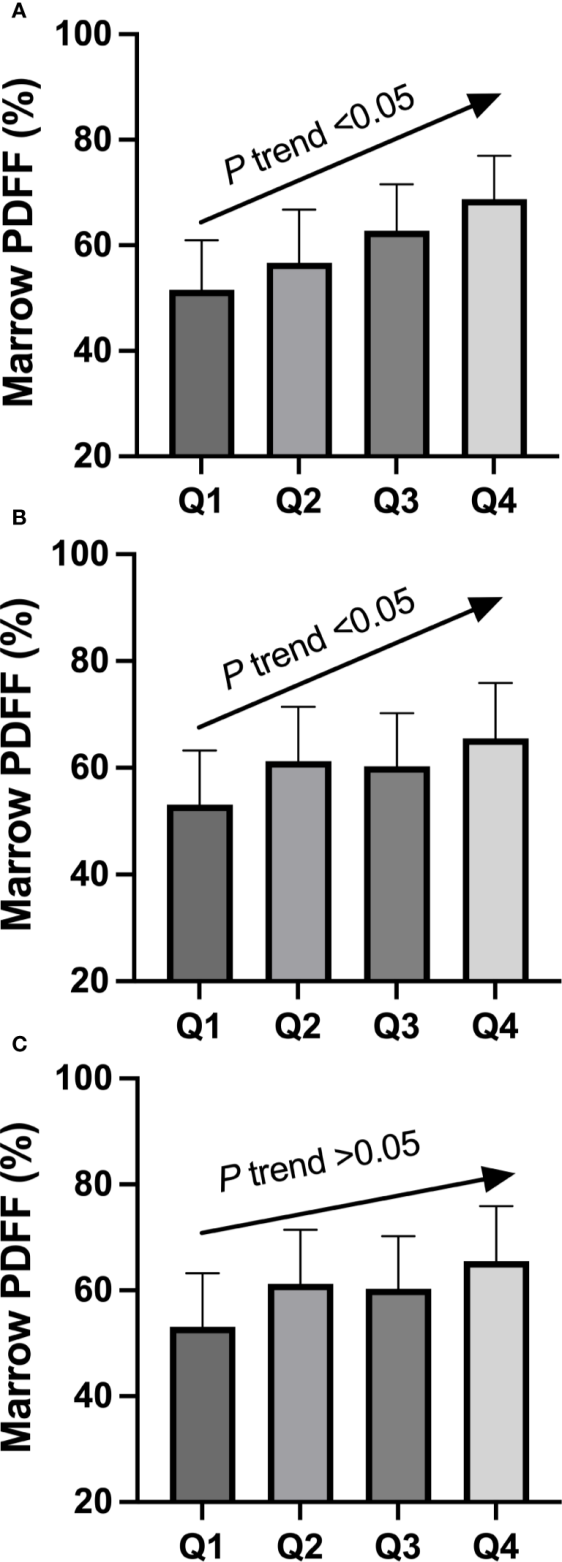
Adjusted mean value of circulating RANKL **(A)**, RANKL/OPG ratio **(B)**, and OPG **(C)** in participants classified according to quartile groups (Q1–Q4) of marrow fat fraction. Mean values were adjusted for age, BMI, physical activity (moderate intensity), total fat mass, android/gynoid ratio, and lean mass. There was a significant linear trend between marrow fat fraction and circulating RANKL quartile groups, as well as between marrow fat fraction and RANKL/OPG ratio quartile groups. OPG, osteoprotegerin; Q, quartile; RANKL, receptor activator of nuclear factor-kappa B ligand.

The demographic data, BMD, body composition, marrow PDFF, and serological characteristics of the study participants are summarized in **Table 1**. The quartiles for circulating RANKL levels in the cohort were as follows: Quartile 1, <112 pg/mL; Quartile 2, 112–157 pg/mL; Quartile 3, 158–196 pg/mL; and Quartile 4, >196 pg/mL. After adjusting for age, BMI, physical activity (moderate intensity), total fat mass, android/gynoid ratio, and lean mass, the least square mean of marrow PDFF was compared across these quartile groups. As shown in **Figure 1**, a significant trend in marrow PDFF was observed across the quartiles of circulating RANKL. Similar trends were observed for the RANKL/OPG ratio, but not OPG levels.

**Table 1:** Baseline characteristics of the participants.

Variables	n = 164
Age, years	68.3 ± 5.9
Time since menopause, years	18.2 ± 6.8
Weight, kg	58.6 ± 6.8
Height, cm	158 ± 5
Body mass index, kg/m^2^	23.6 ± 2.6
Physical activity (moderate intensity), n (%)	39 (20.1%)
Total body fat mass, kg	21.2 ± 4.8
Android fat amount, kg	2.1 ± 0.5
Gynoid fat amount, kg	3.4 ± 0.7
Android/gynoid ratio	0.63 ± 0.13
Total body lean mass, kg	35.0 ± 3.3
Serum calcium, mmol/L	2.40 ± 0.08
Serum phosphorus, mmol/L	1.23 ± 0.13
Serum creatinine, μmol/L	64 (58, 71)
Uric acid, μmol/L	321 (278, 362)
eGFR, mi/min/173m^2^	94.4 ± 7.0
25-hydroxyvitamin D, nmol/L	56.0 (45.4, 72.7)
P1NP, ng/mL	51.7 (41.5, 66.6)
β-type I collagen telopeptides, pg/mL	468 (338, 621)
Osteocalcin, ng/mL	19.7 (16.5, 24.6)
Parathyroid hormone, pg/mL	4.20 (3.28, 5.57)
RANKL, pg/mL	157 (112, 196)
OPG, pg/mL	235 (191, 284)
RANKL/OPG ratio	0.65 (0.51, 0.85)
Femoral neck BMD, g/cm^2^	0.772 (0.696, 0.869)
Total hip BMD, g/cm^2^	0.837 (0.757, 0.925)
Lumbar spine BMD, g/cm^2^	0.945 (0.858, 1.100)
Marrow PDFF, %	59.4 (50.8, 69.1)

Data were expressed as mean ± SD, median (interquartile range) or n (%), where appropriate.

BMD, bone mineral density; eGFR, estimated glomerular filtration rate; OPG, osteoprotegerin; PDFF, proton density fat fraction; P1NP, N-terminal propeptide of type 1 procollagen; RANKL, receptor activator of nuclear factor-kappa B ligand.

### 3.2 Bivariate correlations of circulating RANKL, OPG levels, and their ratio with BMD and marrow PDFF

Age showed a positive correlation with marrow PDFF (*r* = 0.436, *p <*0.001), suggesting it could be a potential confounder. A suggestive correlation was observed between marrow PDFF and β-type I collagen telopeptides (*r* = 0.142, *p* =0.070), but no significant correlations were found with the N-terminal propeptide of type I procollagen (*r* = 0.063, *p* =0.427), parathyroid hormone (*r* = 0.071, *p* =0.369), and osteocalcin (*r* = 0.122, *p* =0.120).

**Figure 2:**
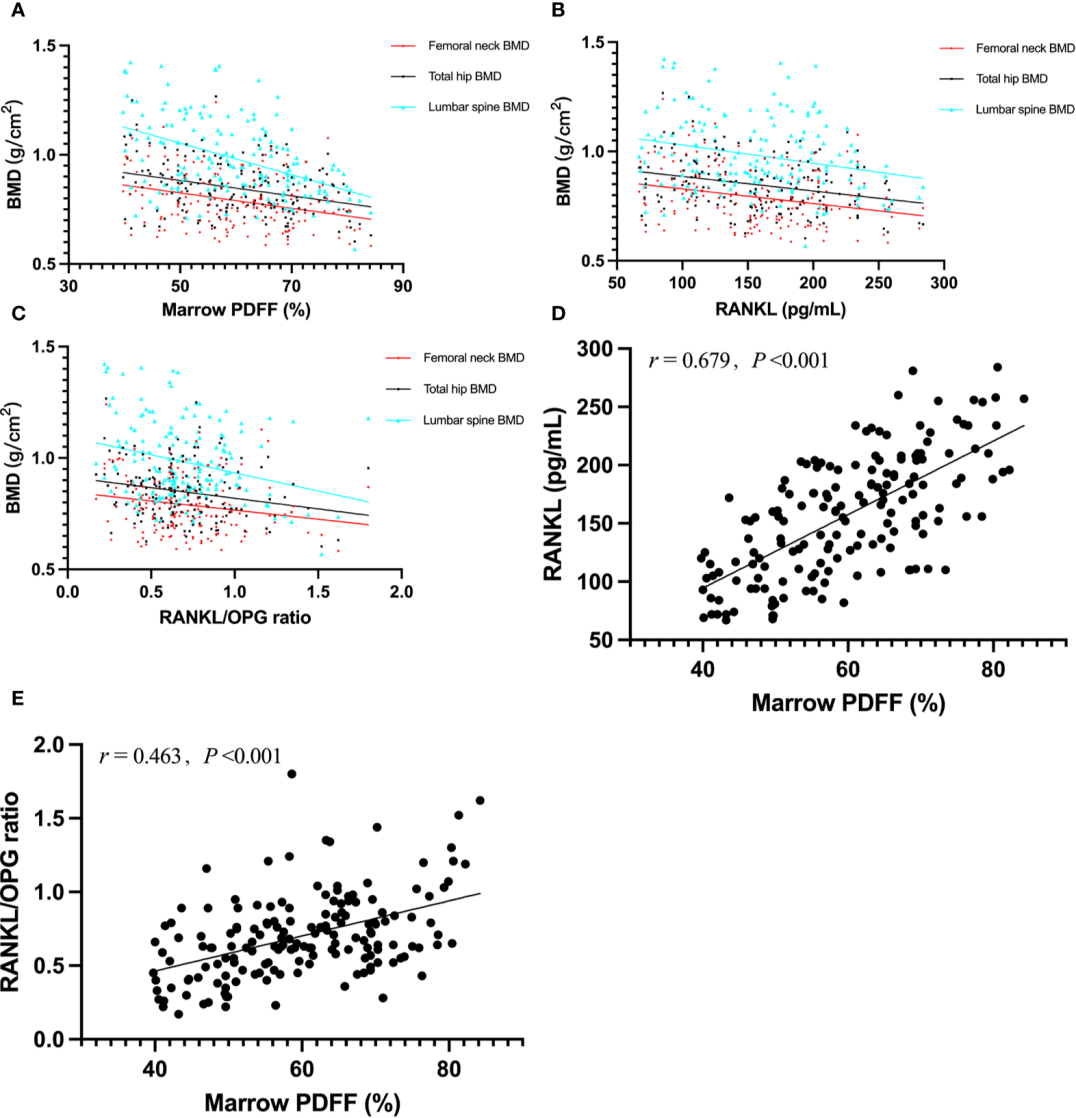
Graphic representation of correlations between marrow PDFF and BMD **(A)**; *r* = –0.462 for lumbar sipne, *r* = –0.317 for total hip and *r* = –0.305 for femoral neck), serum RANKL and BMD **(B)**; *r* = –0.246 for lumbar sipne, *r* = –0.278 for total hip and *r* = –0.269 for femoral neck), RANKL/OPG ratio and BMD **(C)**; *r* = –0.266 for lumbar sipne, *r* = –0.221 for total hip and *r* = –0.183 for femoral neck), marrow PDFF and serum RANKL **(D)**, and marrow PDFF and RANKL/OPG ratio **(E)**. BMD, bone mineral density; PDFF, proton density fat fraction; OPG, osteoprotegerin; RANKL, receptor activator of nuclear factor-kappa B ligand.

The correlations between serum RANKL, OPG, RANKL/OPG ratio, and the parameters of interest are shown in **Table 2**. In terms of BMD, we found an inverse correlation between marrow PDFF and BMD at the lumbar spine (*r* = −0.462), total hip (*r* = −0.317), and femoral neck (*r* = −0.305, all *p* < 0.001). Serum RANKL levels and RANKL/OPG ratio were found to be negatively correlated with BMD at the lumbar spine, total hip, and femoral neck, and positively associated with marrow PDFF (all *p* < 0.05) (**Figure 2**). However, circulating OPG levels were not associated with marrow PDFF or BMD at any skeletal site, except for a mild positive correlation between circulating OPG and lumbar spine BMD.

**Table 2:** Correlation coefficients among the RANKL, OPG, RANKL/OPG ratio, BMD, and marrow PDFF.

	Femoral neck BMD	Total hip BMD	Lumbar spine BMD	OPG	RANKL	RANKL/OPG ratio	Marrow PDFF
Femoral neck BMD	1.000	**0.906**	**0.528**	0.025	**−0.269**	**−0.183**	**−0.305**
Total hip BMD	**0.906**	1.000	**0.618**	0.052	**−0.278**	**−0.221**	**−0.317**
Lumbar spine BMD	**0.528**	**0.618**	1.000	**0.182**	**−0.246**	**−0.266**	**−0.462**
OPG	0.025	0.052	**0.182**	1.000	**0.167**	**−0.574**	0.074
RANKL	**−0.269**	**−0.278**	**−0.246**	**0.167**	1.000	**0.639**	**0.679**
RANKL/OPG ratio	**−0.183**	**−0.221**	**−0.266**	**−0.574**	**0.639**	1.000	**0.463**
Marrow PDFF	**−0.305**	**−0.317**	**−0.462**	0.074	**0.679**	**0.463**	1.000

BMD, bone mineral density; PDFF, proton density fat fraction; OPG, osteoprotegerin; RANKL, receptor activator of nuclear factor-kappa B ligand.

Significant p-values are depicted in bold.

### 3.3 Vertebral marrow fat fraction is independently associated with circulating soluble RANKL levels

Multiple regression analysis was performed to assess the influences of BMD and PDFF on serum RANKL, OPG, and the RANKL/OPG ratio, with results presented in **Table 3**. The analysis showed that marrow PDFF and BMD were significant contributing factors to serum soluble RANKL levels (β = 3.15, 95% CI 2.60 to 3.70 for PDFF; β = −0.200, 95% CI −0.348 to −0.051 for vertebral BMD; β = −0.383, 95% CI −0.589 to −0.177 for total hip BMD; and β =−0.393, 95% CI −0.598 to −0.188 for femoral neck BMD, all *p* < 0.01) in postmenopausal females, after adjusting for age, BMI, physical activity (moderate intensity), total fat mass, android/gynoid ratio, and lean mass. This association remained significant for marrow PDFF even after additional adjustment for BMD (β = 1.34, 95% CI 1.10 to 1.58, *p* < 0.001). Similar results were obtained for the RANKL/OPG ratio. Conversely, marrow PDFF and BMD did not significantly affect OPG levels (**Table 3**).

**Table 3:** Multiple linear regression analysis: Marrow PDFF, BMD at the lumber spine, total hip, and femoral neck, respectively as dependent parameters.

	Log (RANKL)		Log (OPG)		Log (RANKL/OPG ratio)	
	β (95% CI)	*p*-values	β (95% CI)	*p*-values	β (95% CI)	*p*-values
Model 1						
Lumbar spine BMD	−0.200 (−0.348 to −0.051)	0.009	0.039 (−0.102 to 0.180)	0.582	−0.289 (−0.463 to −0.116)	0.001
Femoral neck BMD	−0.393 (−0.598 to −0.188)	<0.001	−0.003 (−0.202 to 0.196)	0.977	−0.362 (−0.610 to −0.115)	0.004
Total hip BMD	−0.383 (−0.589 to −0.177)	<0.001	−0.011 (−0.220 to 0.199)	0.921	−0.402 (−0.661 to −0.143)	0.003
Marrow PDFF	3.15 (2.60 to 3.70)	<0.001	0.001 (−0.001 to 0.003)	0.436	1.07 (0.756 to 1.38)	<0.001
Model 2						
Lumbar spine BMD	0.080 (−0.042 to 0.203)	0.197	0.077 (−0.079 to 0.232)	0.332	−0.080 (−0.253 to 0.093)	0.364
Marrow PDFF	1.34 (1.10 to 1.58)	<0.001	0.001 (−0.001 to 0.004)	0.265	1.00 (0.662 to 1.34)	<0.001

BMD, bone mineral density; CI, confidence interval; PDFF, proton density fat fraction; OPG, osteoprotegerin; RANKL, receptor activator of nuclear factor-kappa B ligand.

Model 1: p-values were adjusted for age, body mass index, physical activity, total fat mass, android/gynoid ratio, and lean mass.

Model 2: p-values were adjusted for age, body mass index, physical activity, total fat mass, android/gynoid ratio, lean mass, marrow PDFF (for lumbar spine BMD) or lumbar spine BMD (for marrow PDFF).

## 4 Discussion

In this study, multiple regression analysis was used to assess the influence of BMD at various skeletal sites on RANKL, OPG, and the RANKL/OPG ratio. The fingings revealed that BMD significantly impacted circulating soluble RANKL but not OPG in postmenopausal females, which aligns partially with existing literature. Previous research has shown inconsistent effects of circulating RANKL and OPG on serum markers of bone turnover and BMD. For instance, some studies have reported elevated levels of soluble RANKL in ovariectomized mice (1), whereas others found no difference in RANKL levels between premenopausal and postmenopausal females, despite higher RANKL expression in mesenchymal stem cells of postmenopausal females (9), or between individuals with normal and low bone mass (8). Additionally, some studies have indicated a significant negative association between serum RANKL and BMD (11). Similarly, the relationship between circulating OPG levels and BMD has shown mixed results in various studies of postmenopausal females (6–8, 10, 11, 26). Interestingly, Nabipour et al. (10) found that serum levels of RANKL and OPG were independent determinants of BMD. However, Mezquita-Raya et al. (26) reported that OPG, but not RANKL, correlated with vertebral fractures and osteoporosis in postmenopausal females. In that study, serum RANKL concentrations were undetectable in a substantial proportion of women (54.9%). Among the detectable levels, most were exceedingly low, likely representing assay noise. Eghbali-Fatourechi et al. (9) corroborated our findings, suggesting that the upregulation of RANKL on bone marrow cells is a crucial factor in the increased bone resorption associated with estrogen deficiency. These discrepancies may be attributed to differences in study design, sample size, methodology, statistical analysis, and other unknown factors. They might also suggest that serum RANKL and OPG are not reflective of the biological effects of these molecules at the tissue level.

The age-related increase in fatty marrow typically surpasses the reduction in cellular marrow, likely because fatty marrow progressively replaces both hematopoietic tissue and bone (27). Recent studies have highlighted the role of marrow adipose tissue in regulating energy metabolism and bone homeostasis. Excessive fat content in the marrow is associated with metabolic dysfunction, disrupting the balance among osteoblast, osteoclast, and adipocyte activity that maintains bone mass. Increased marrow fat content often correlates with decreased bone mass, as observed in previous aging studies (12, 28) and confirmed in our study of postmenopausal females. In our study, no significant correlation was found between vertebral marrow PDFF and bone turnover biomarkers, such as parathyroid hormone, β-type I collagen telopeptides, osteocalcin, and the N-terminal propeptide of type 1 procollagen. These results align with earlier studies indicating that vitamin D, calcium, and parathyroid hormone levels do not correlate with vertebral marrow fat content in postmenopausal women with type 2 diabetes mellitus, patients with chronic kidney disease, or healthy controls (29–31).

Regarding marrow adipose tissue, the relationship between circulating soluble RANKL and marrow fat content aligned with our expectations. One key finding was that marrow adipose tissue was a determinant of variation in circulating soluble RANKL in postmenopausal females. This suggests that the secretion of soluble RANKL is, to some extent, specific to marrow adipose tissue and that serum concentrations may reflect intracellular concentrations or activity within the bone microenvironment. Increased marrow adipose tissue is often accompanied by bone mass loss due to elevated bone resorption. A prior study analyzed RANKL gene expressions in peripheral fat depots, including inguinal, epididymal, and interscapular adipose depots. Compared to marrow fat tissue, RANKL expression was virtually undetectable in these peripheral adipose depots (32), indicating the capability of marrow fat tissue, but not other adipose depots, to secrete RANKL. Primary human bone marrow adipocytes play a favorable role in osteoclast differentiation and function by expressing the pro-osteoclastogenic factor RANKL (33). In murine studies, aging significantly increased stromal/osteoblastic cell-induced osteoclastogenesis. Marrow adipocytes with osteogenic and adipogenic features can secrete RANKL and impact bone resorption. During marrow adipogenesis, RANKL expression was induced through the action of C/EBPβ and/or C/EBPδ, and RANKL-positive pre-adipocytes increased in the bone marrow of aged mice, concomitantly with a downregulation of osteoprotegerin (22). Interestingly, the lack of soluble RANKL did not influence bone loss caused by estrogen deficiency (4).

This study has several limitations. Firstly, the cross-sectional design limits our ability to establish direct causal relationships between variables. Secondly, the findings may not be generalizable to younger age groups or men, as the study focused solely on postmenopausal females. Thirdly, despite the statistical analysis accounting for numerous covariates, certain unconsidered factors, such as dietary influences or comorbidities, cannot be completely ruled out. Lastly, the measurement of circulating RANKL levels may not fully reflect tissue levels. Further investigation is needed to determine the direct link between marrow fat content and soluble RANKL using experimental studies.

## 5 Conclusion

In this observed sample, marrow fat content was negatively correlated with BMD at various skeletal sites in postmenopausal females, and marrow adipose tissue was identified as a determinant of variation in circulating soluble RANKL. The potential direct link between marrow fat content and soluble RANKL should be further explored through experimental studies. Additional research is necessary to clarify whether the increased bone resorption observed in pathological conditions like osteoporosis is driven by RANKL originating from marrow adipose tissue rather than from the bone tissue itself.

## Data Availability

The raw data supporting the conclusions of this article will be made available by the authors, without undue reservation.
